# Supplementary material for: Tetrachromatic vision-inspired neuromorphic sensors with ultraweak ultraviolet detection
Source: Nat Commun. 2023 Apr 21;14:2281. doi: 10.1038/s41467-023-37973-0 (PMC10121588; doi:10.1038/s41467-023-37973-0)
Supplement: Supplementary file 1 — Supplementary Information [file 41467_2023_37973_MOESM1_ESM.pdf]

Supplementary Information

Tetrachromatic vision-inspired neuromorphic sensors with ultraweak ultraviolet detection

Ting Jiang[1,#], Yiru Wang[2,#], Yingshuang Zheng[1], Le Wang[2], Xiang He[2], Liqiang Li[1,3], Yunfeng Deng[4], Huanli Dong[5], Hongkun Tian[6*], Yanhou Geng[4], Linghai Xie[2], Yong Lei[7], Haifeng Ling[2*], Deyang Ji[1,3*]and Wenping Hu[3,8,9]

[1]Tianjin Key Laboratory of Molecular Optoelectronic Sciences, Department of Chemistry, Institute of Molecular Aggregation Science, Tianjin University, Tianjin 300072, China.

[2]State Key Laboratory of Organic Electronics and Information Displays & Institute of Advanced Materials (IAM), Nanjing University of Posts & Telecommunications, Nanjing 210023, China.

[3]Haihe Laboratory of Sustainable Chemical Transformations, Tianjin 300192, China.

[4]School of Materials Science and Engineering, Tianjin University, Tianjin 300072, China.

[5]Beijing National Laboratory for Molecular Sciences, Key Laboratory of Organic Solids, Institute of Chemistry, Chinese Academy of Sciences, Beijing 100190, China.

[6]State Key Laboratory of Polymer Physics and Chemistry, Changchun Institute of Applied Chemistry, Chinese Academy of Sciences, Changchun 130022, China.

[7]Fachgebiet Angewandte Nanophysik, Technische Universität Ilmenau, Ilmenau 98693, Germany

[8]Tianjin Key Laboratory of Molecular Optoelectronic Sciences, Department of Chemistry, School of Science, Tianjin University. Collaborative Innovation Center of Chemical Science and Engineering, Tianjin 300072, China.

[9]Joint School of National University of Singapore and Tianjin University, Fuzhou 350207, China.

[#]These authors contributed equally: Ting Jiang, Yiru Wang.

E-mail: jideyang@tju.edu.cn; iamhfling@njupt.edu.cn; hktian@ciac.ac.cn

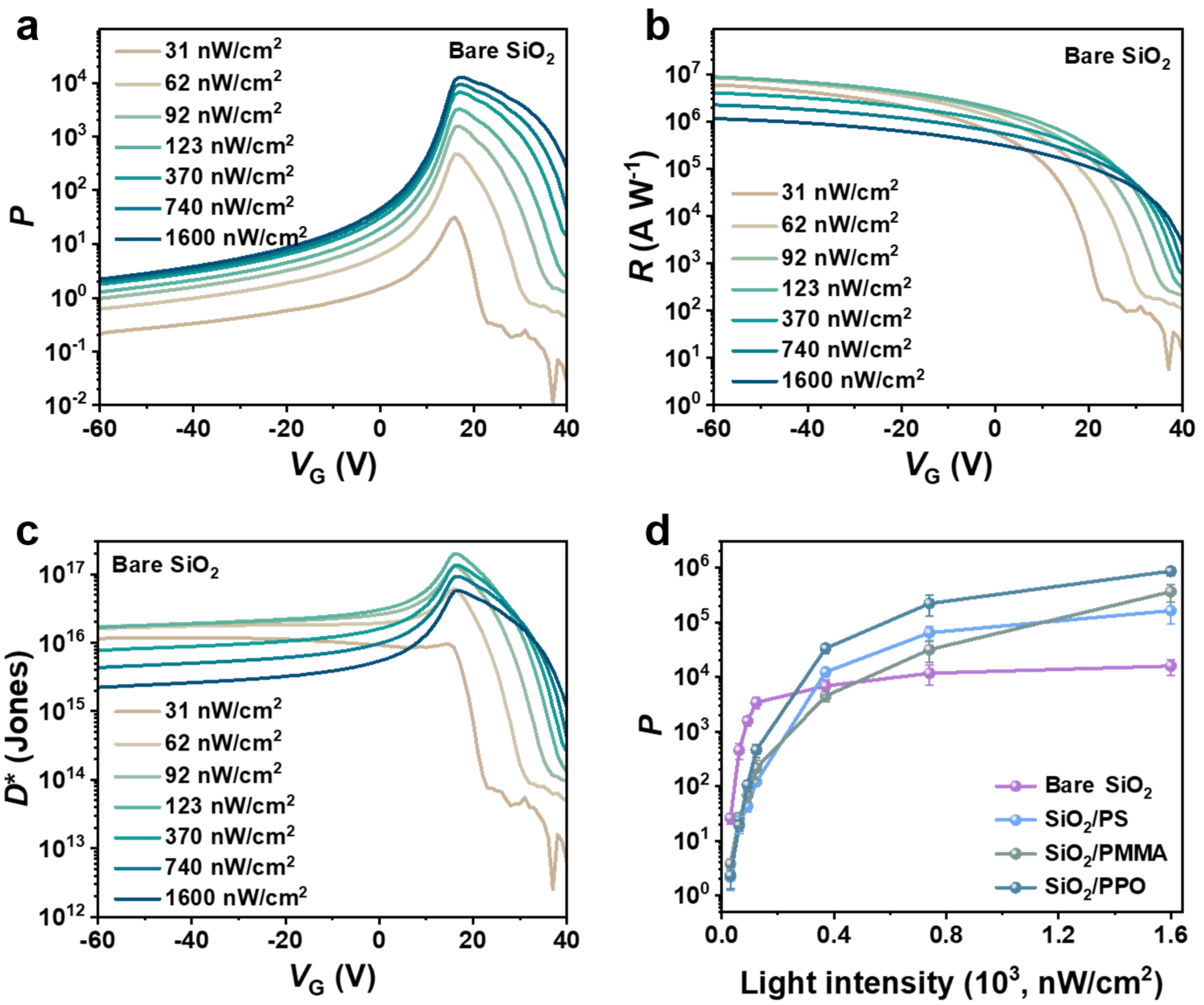

Figure S1. a~c P, R and D^* of the phototransistor based on bare SiO_2 dielectric at various illumination intensities. **d** P_{max} in different interface as a function of illumination intensity. The values and error bars were the mean value and standard deviation obtained with 10 OPT devices.

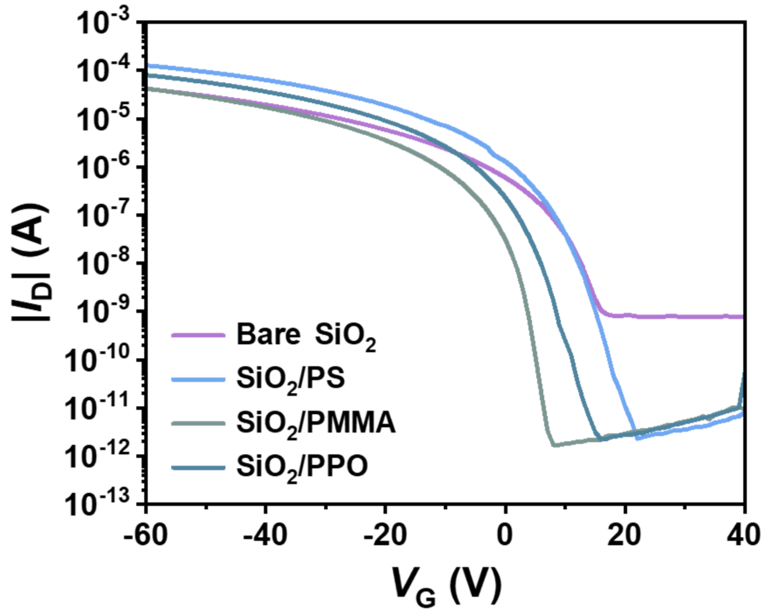

Figure S2. The transfer curves of different interface.

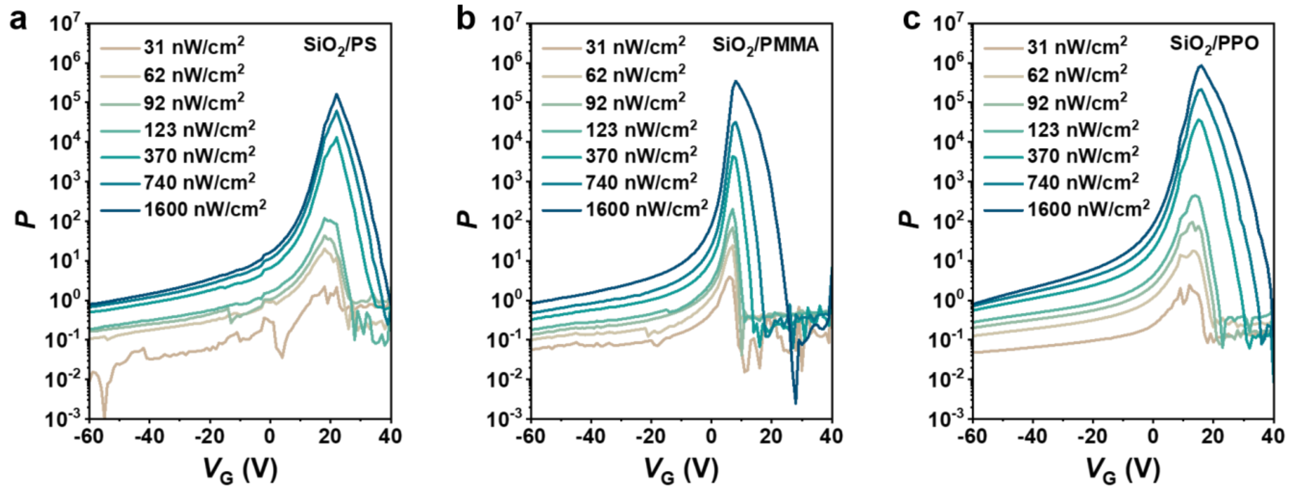

Figure S3. The photosensitivity (P) of the phototransistor based on **a** SiO_2/PS; **b** SiO_2/PMMA; **c** SiO_2/PPO.

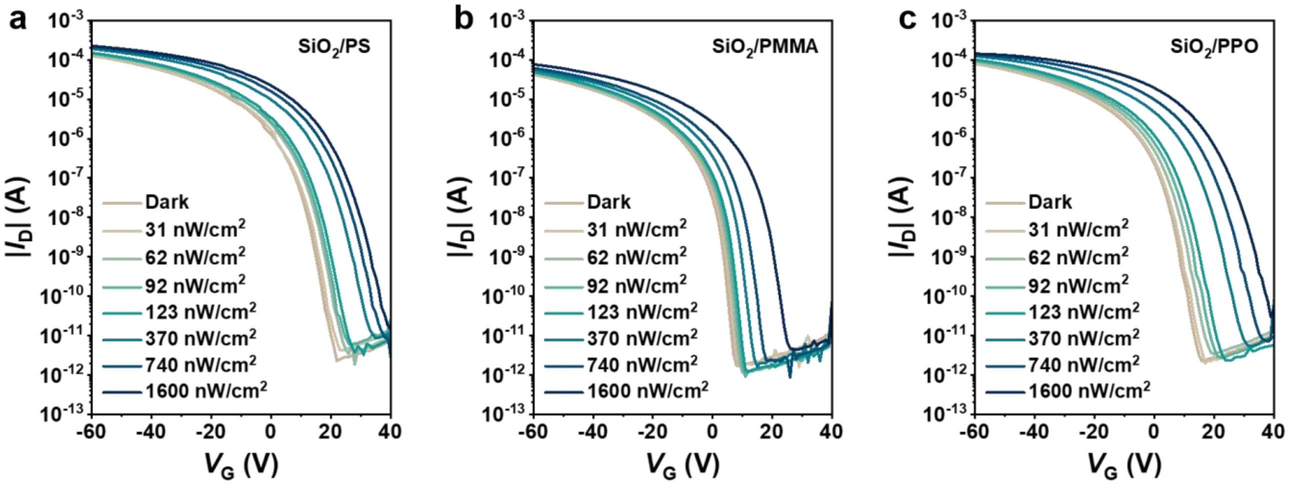

Figure S4. The transfer curves of the UV-sensitive OPTs based on **a** SiO_2/PS; **b** SiO_2/PMMA; **c** SiO_2/PPO.

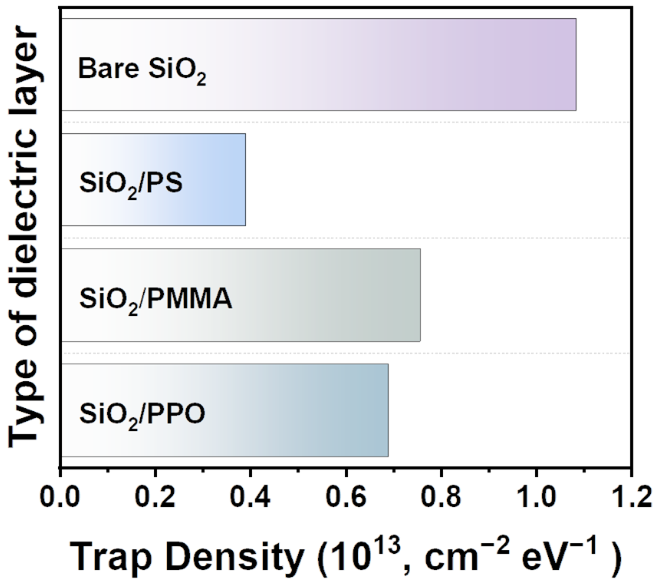

Figure S5. Trap density with bare SiO_2, SiO_2/PS, SiO_2/PMMA, SiO_2/PPO.

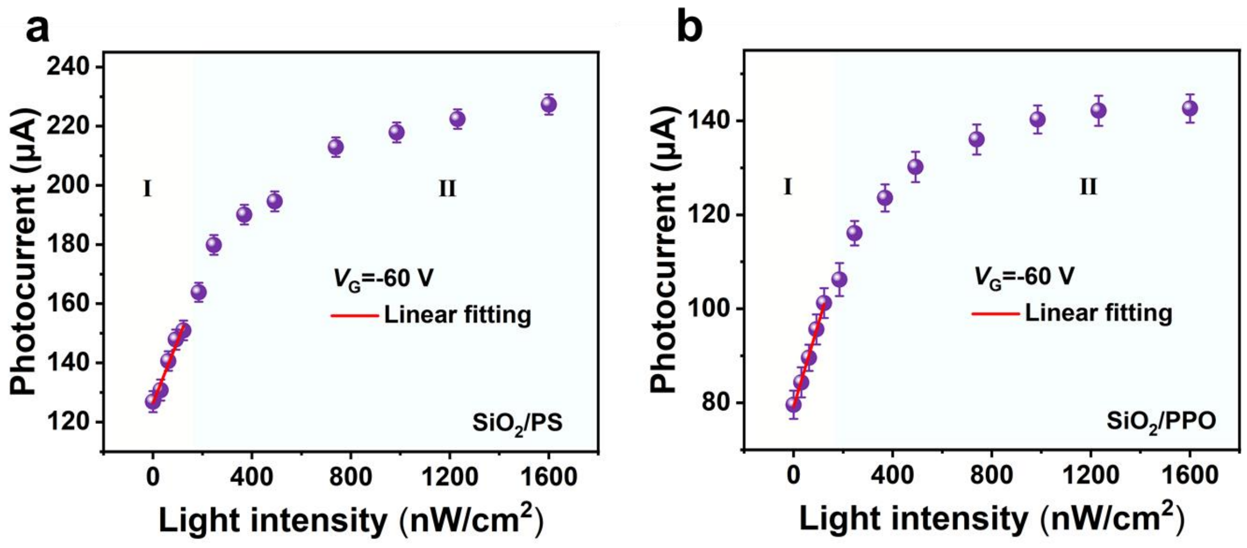

Figure S6. The dependence of photocurrent on light intensity based on **a** SiO_2/PS; **b** SiO_2/PPO. The values and error bars were the mean value and standard deviation obtained with 10 OPT devices.

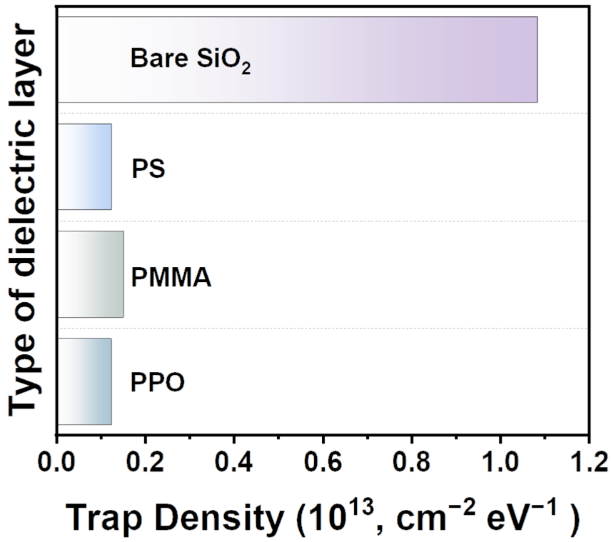

Figure S7. Trap density with bare SiO_2, PS, PMMA, PPO.

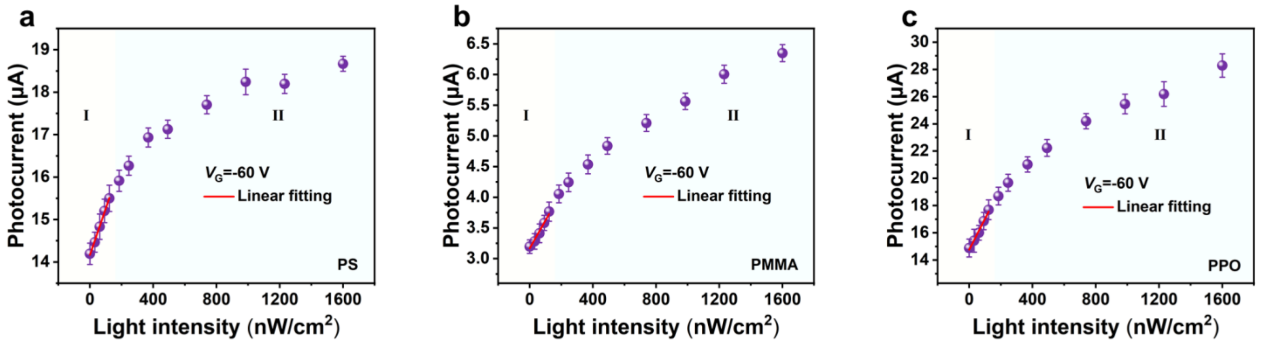

Figure S8. The dependence of photocurrent on light intensity based on **a** PS, **b** PMMA and **c** PPO. The values and error bars were the mean value and standard deviation obtained with 10 OPT devices.

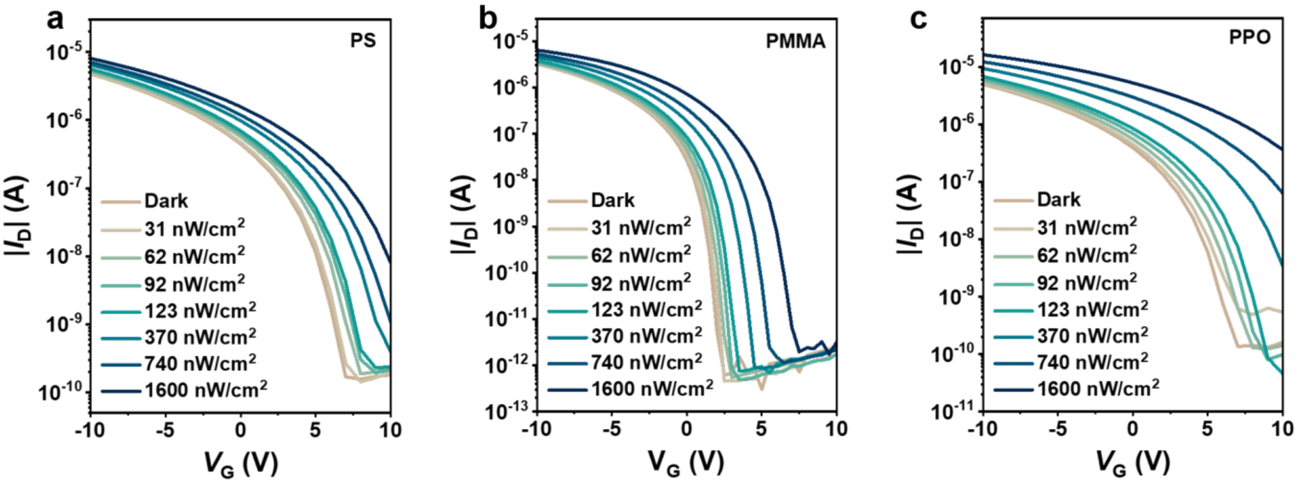

Figure S9. The transfer curves of the UV-sensitive OPTs based on **a** PS, **b** PMMA and **c** PPO.

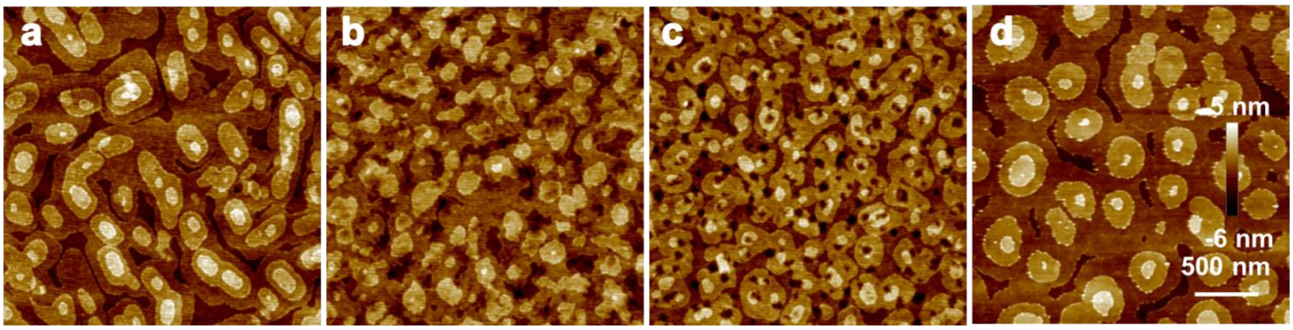

Figure S10. 3 μm×3 μm AFM images of BTBTT6-syn films on different dielectric surfaces, **a** Bare SiO_2, **b** SiO_2/PS, **c** SiO_2/PMMA and **d** SiO_2/PPO.

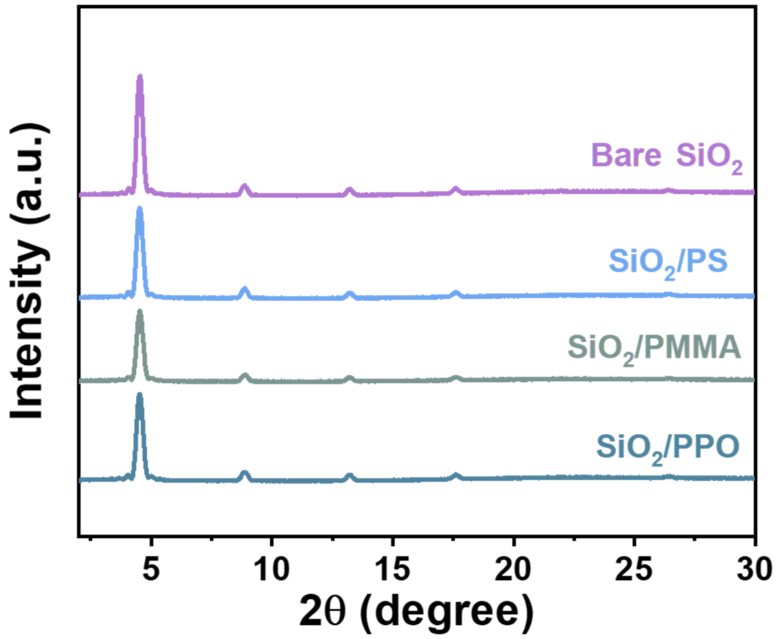

Figure S11. XRD patterns of BTBTT6-syn films vacuum deposited on bare SiO_2, SiO_2/PS, SiO_2/PMMA, SiO_2/PPO.

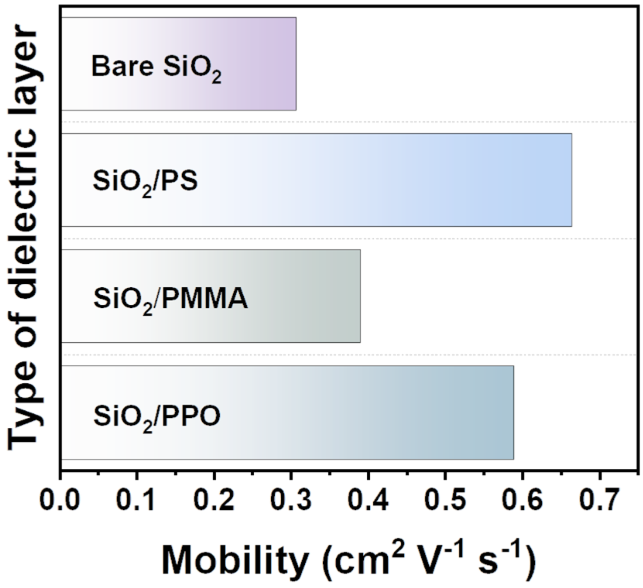

Figure S12. Mobility with bare SiO_2, SiO_2/PS, SiO_2/PMMA, SiO_2/PPO.

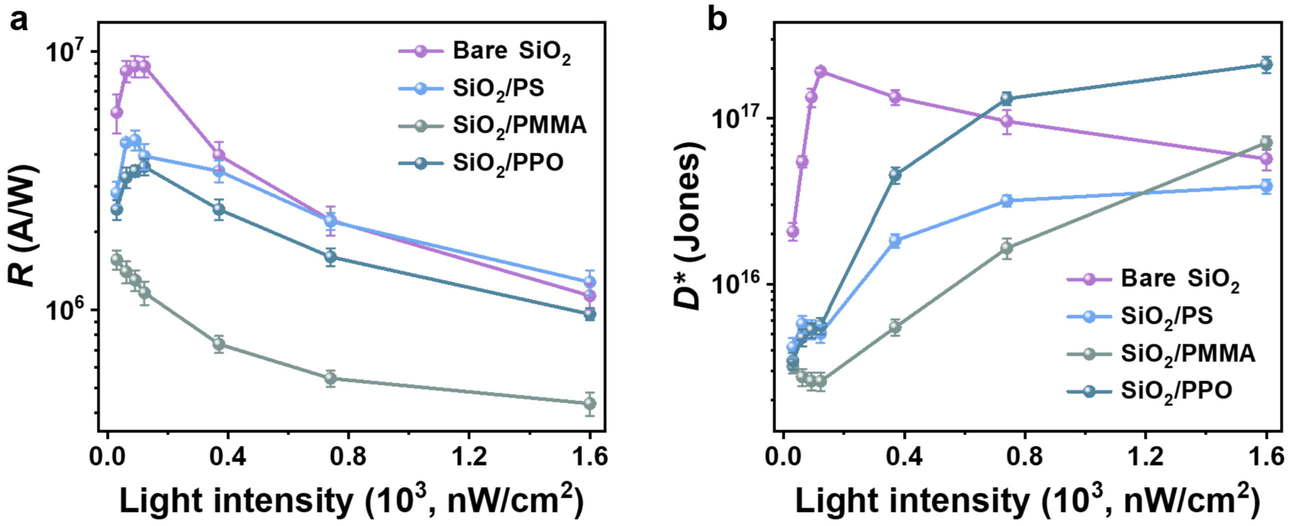

Figure S13. **a** R in different interface as a function of illumination intensity. **b** D^* in different interface as a function of illumination intensity. The values and error bars were the mean value and standard deviation obtained with 10 OPT devices.

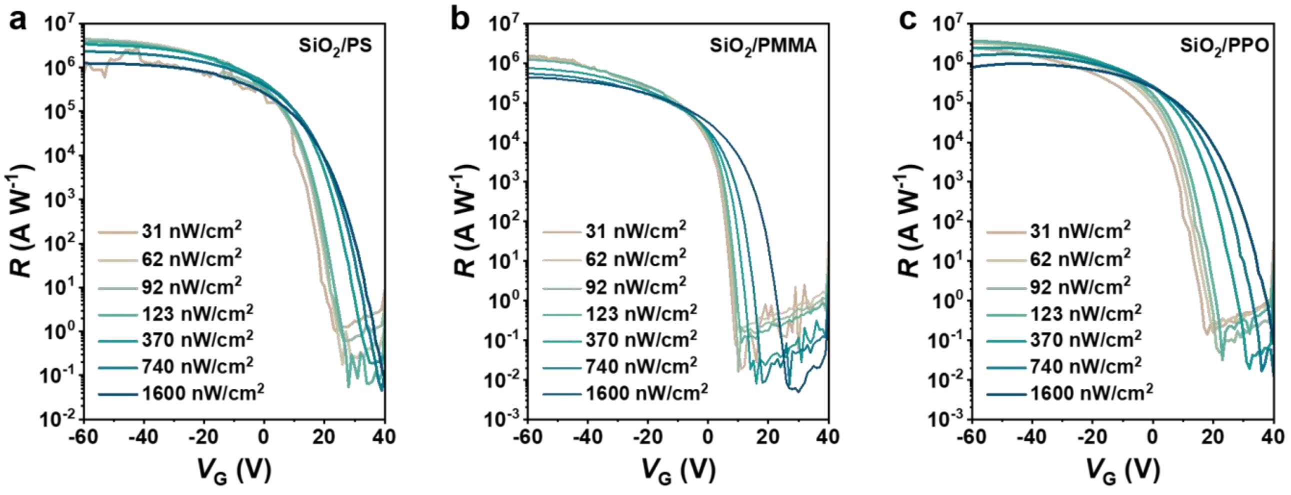

Figure S14. The photoresponsivity (R) of the phototransistor based on **a** SiO_2/PS; **b** SiO_2/PMMA; **c** SiO_2/PPO.

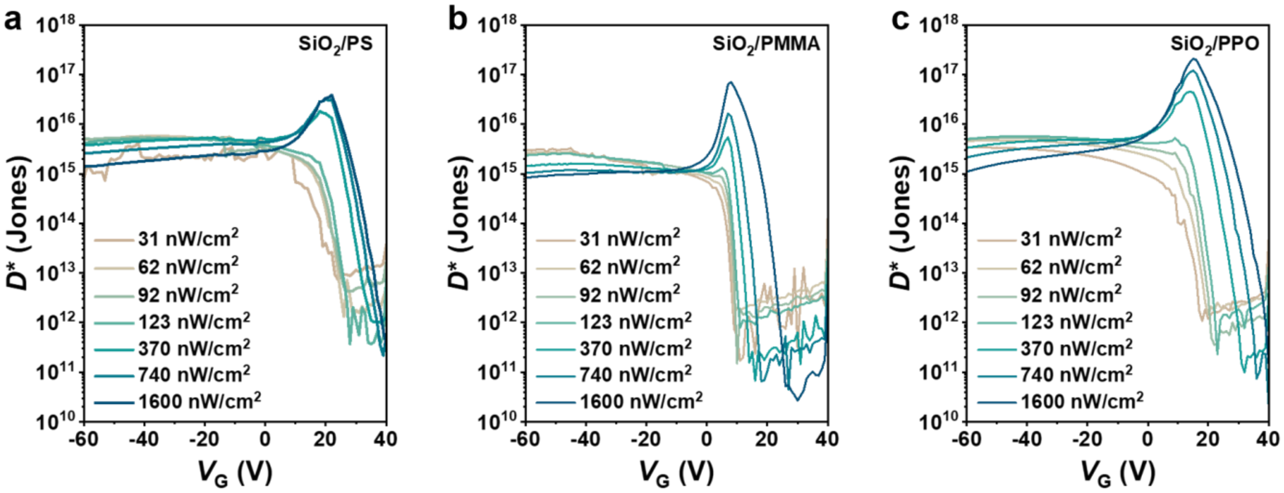

Figure S15. The detectivity (D^{*}) of the phototransistor based on **a** SiO_2/PS; **b** SiO_2/PMMA; **c** SiO_2/PPO.

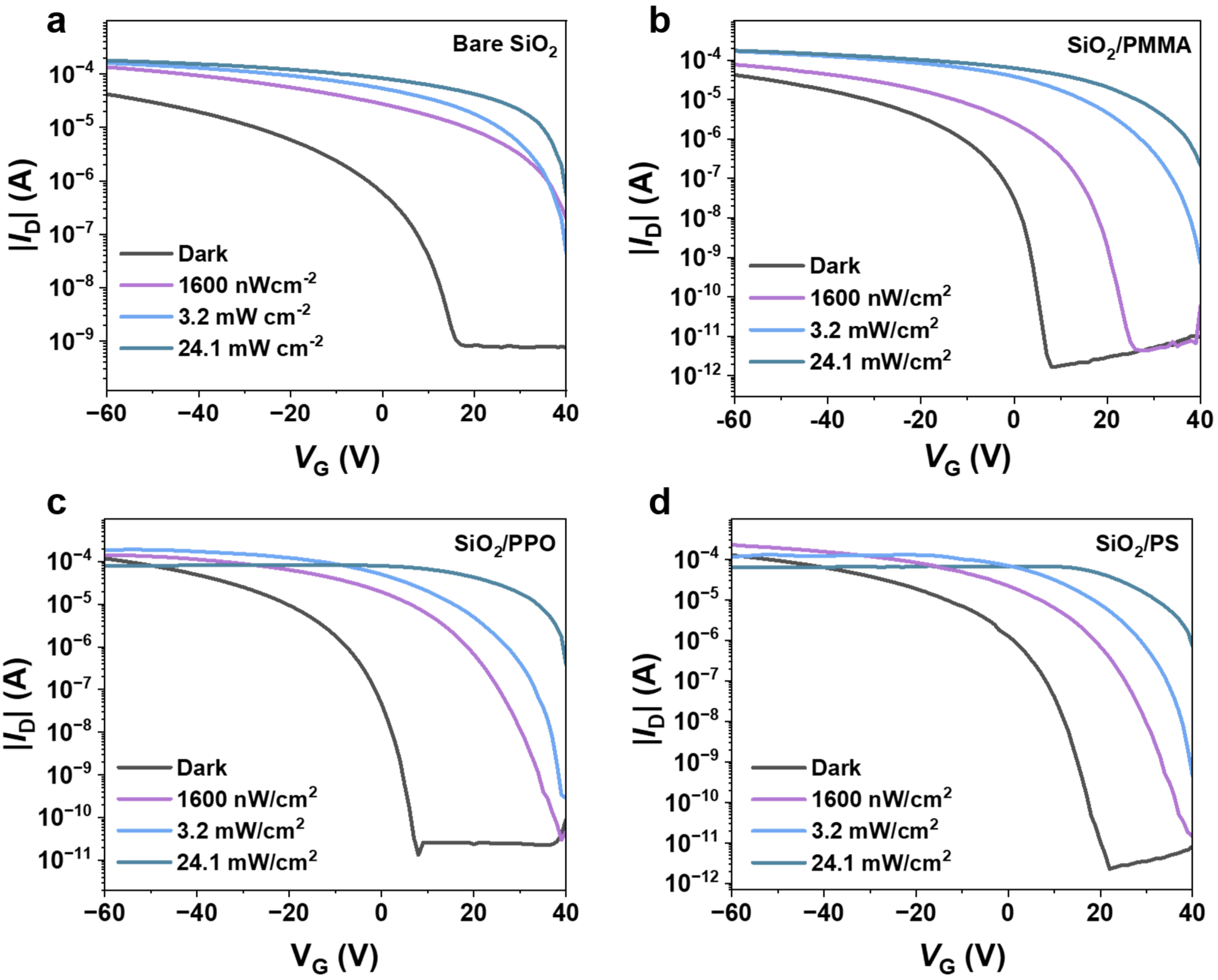

Figure S16. The transfer curves of the UV-sensitive OPTs based on **a** SiO_2, **b** SiO_2/PMMA, **c** SiO_2/PPO, **d** SiO_2/PS.

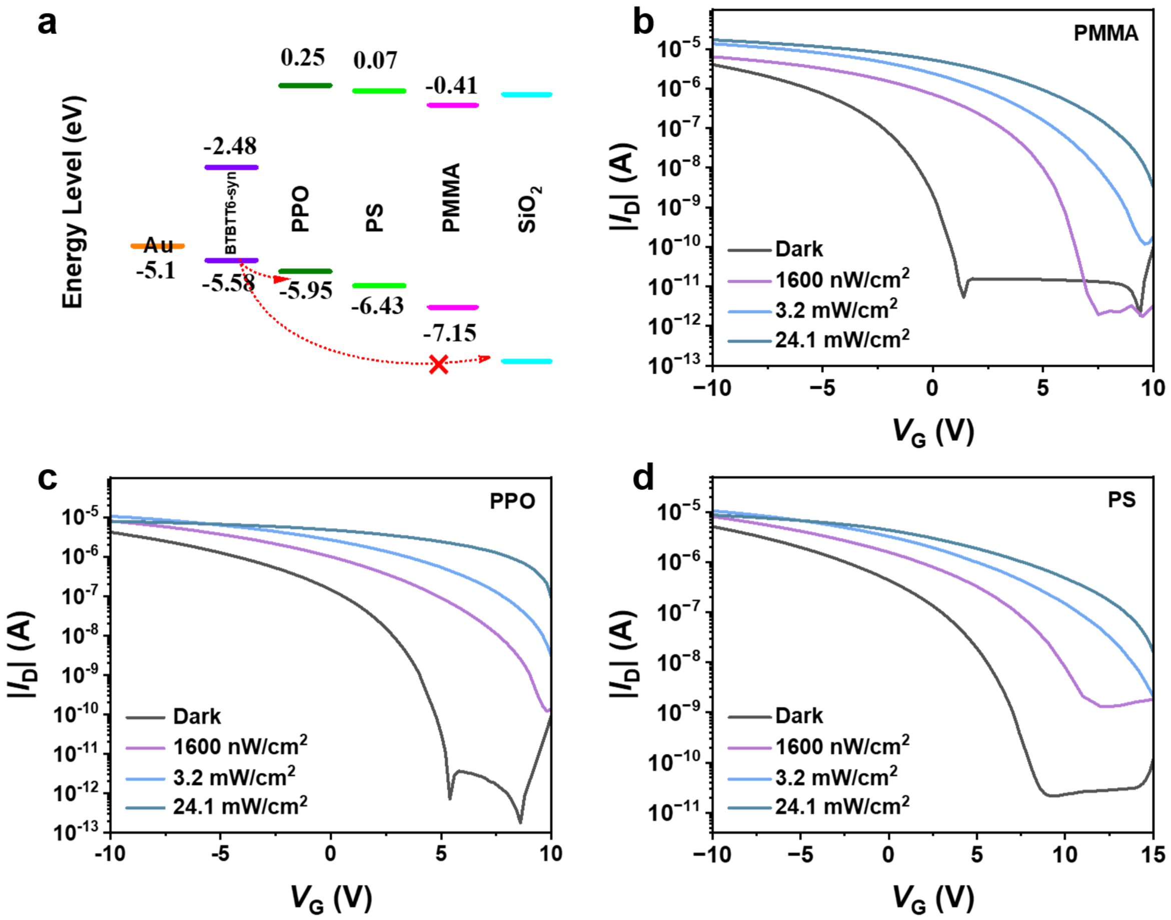

Figure S17. **a** Schematic energy band structures corresponding to the hole transport. The transfer curves of the UV-sensitive OPTs based on **b** PMMA, **c** PPO and **d** PS.

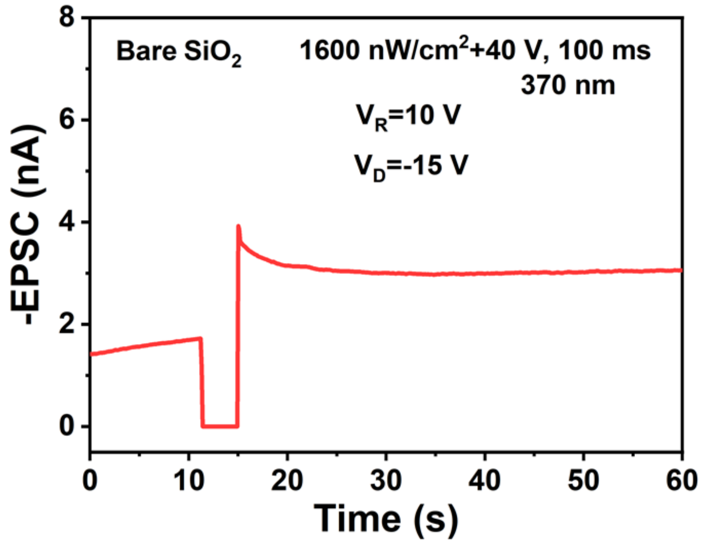

Figure S18. EPSC of the bare SiO_2 device triggered by a short light pulse of 100 ms. V_D and V_R represent for the drain voltage and reading gate voltage, respectively.

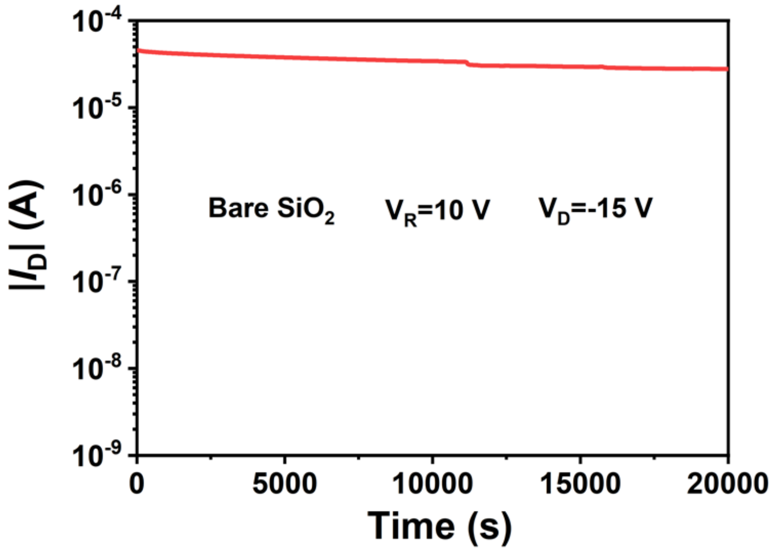

Figure S19. The retention property of the bare SiO_2 device. V_D and V_R represent for the drain voltage and reading gate voltage, respectively.

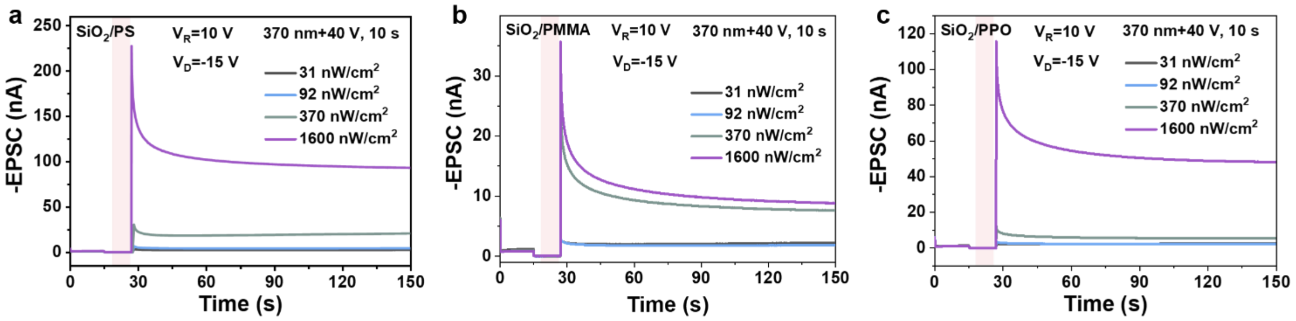

Figure S20. EPSC of the devices with different buffer layers triggered by various illumination intensities **a** SiO_2/PS; **b** SiO_2/PMMA; **c** SiO_2/PPO. The light pink shading regions represent the duration of the UV light stimuli. V_D and V_R represent for the drain voltage and reading gate voltage, respectively.

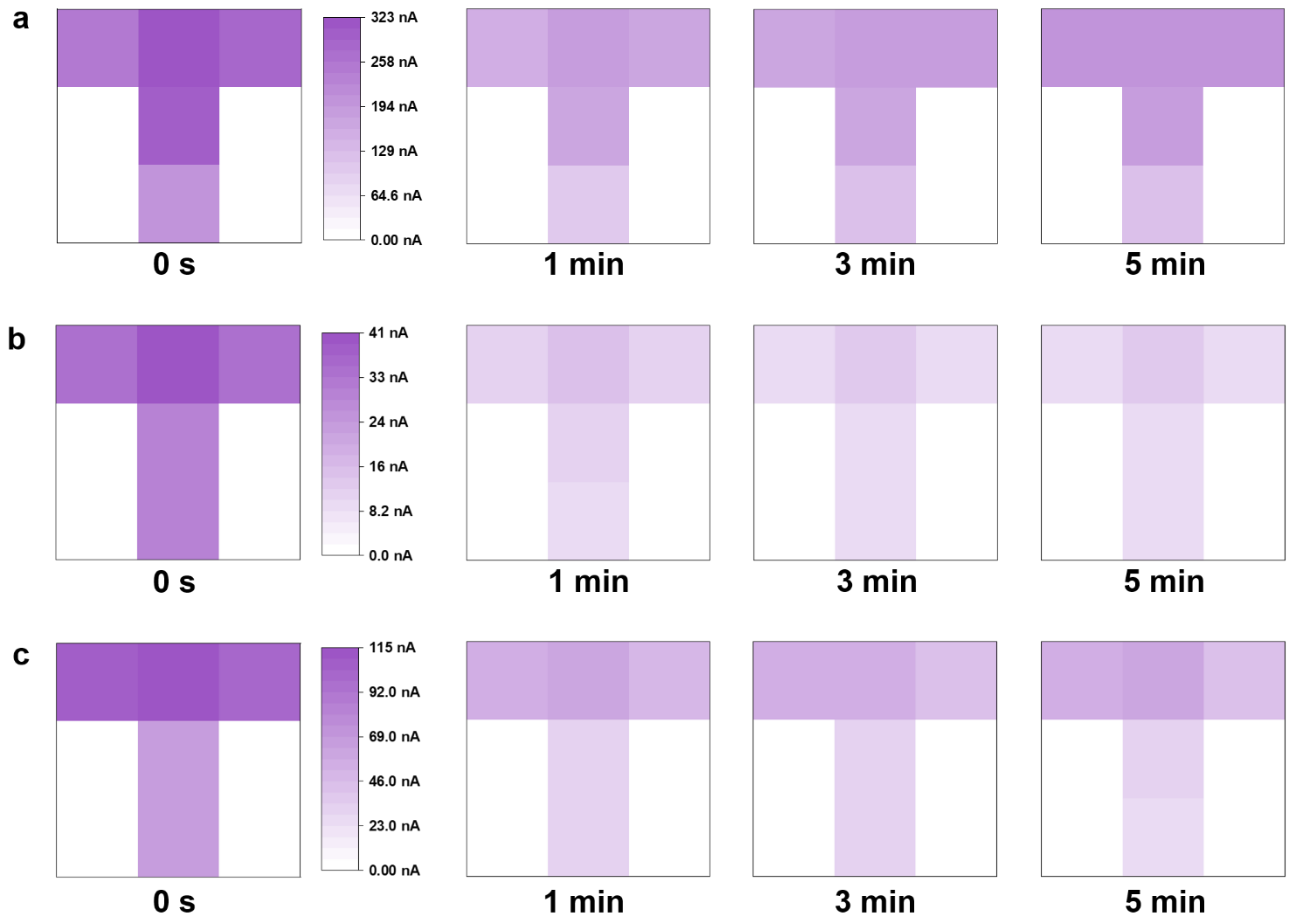

Figure S21. The image memorization property of the devices with different buffer layers **a** SiO_2/PS; **b** SiO_2/PMMA; **c** SiO_2/PPO.

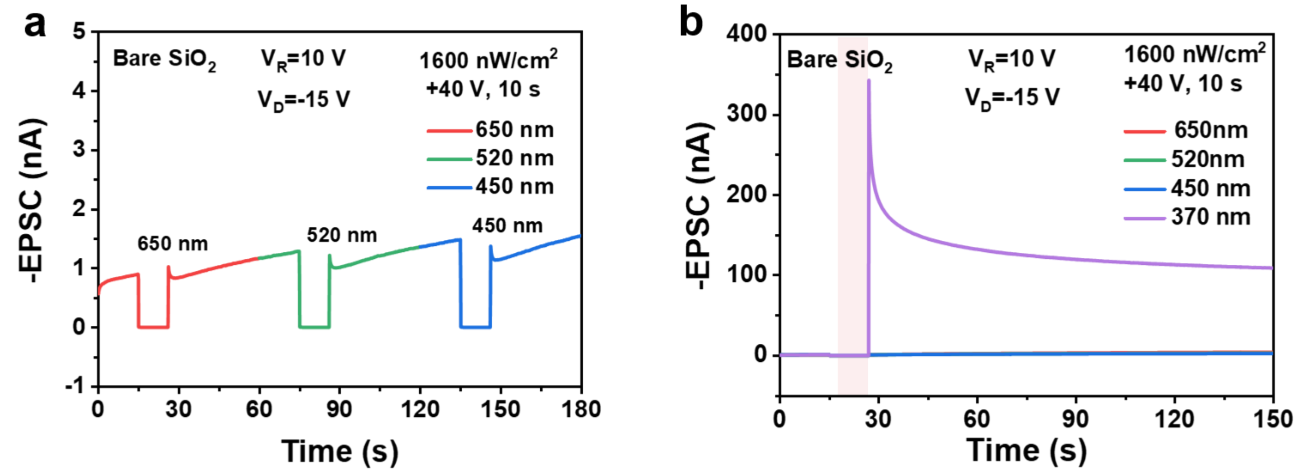

Figure S22. EPSC of bare SiO_2 device triggered by various wavelengths illumination **a** RGB; **b** RGB and UV. The light pink shading region represents the duration of the different light stimuli. V_D and V_R represent for the drain voltage and reading gate voltage, respectively.

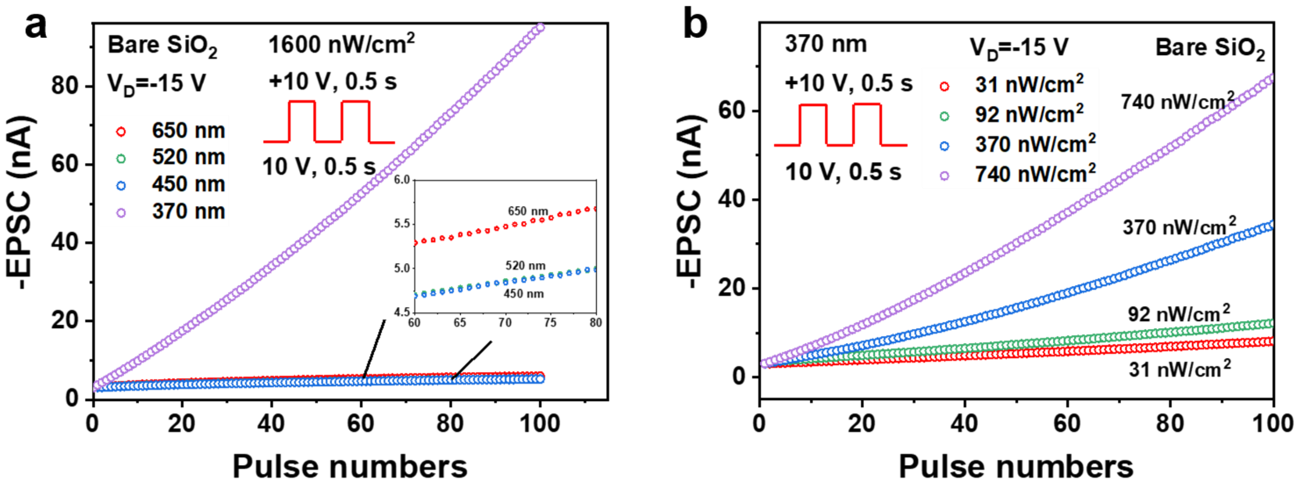

Figure S23. LTP of bare SiO_2 device triggered by various **a** wavelengths; **b** illumination intensities. V_D represents for the drain voltage.

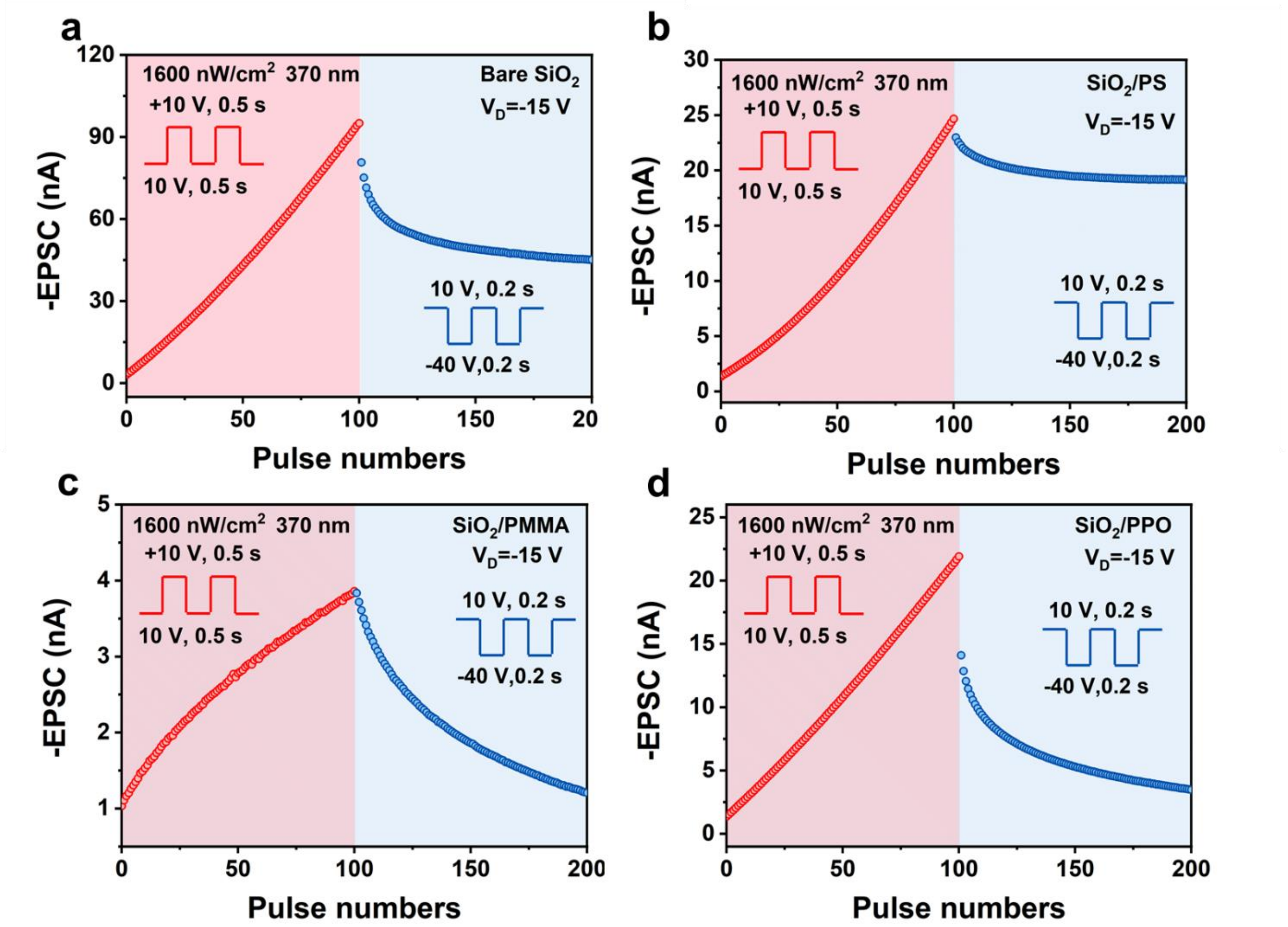

Figure S24. LTP/LTD curves of the devices with different buffer layers **a** bare SiO_2; **b** SiO_2/PS; **c** SiO_2/PMMA; **d** SiO_2/PPO. The pink and blue shading regions represent for the LTP and LTD regions, respectively. V_D represents for the drain voltage.

Table S1. Comparison of current work with representative phototransistors.

Response material	Detection limit (mw cm^{-2})	Wavelength (nm)	V_D (V)	Rise time	Decay time	P_{Max}	R_{Max} (A W^{-1})	D^*_{Max} (Jones)	Growth mode	Ref.
DFD+TMTES-P	0.078	365	-60	N/A	N/A	5.3×10^3	5.3×10^3	2.78×10^{14}	cocrystal	1
C8-BTBT+$AgBiS_2$ QD	0.0038	365	-40	0.23 s	0.82 s	1.5×10^3	20	4×10^{13}	hybrid films (spin-coating)	2
BBDTE	0.037	380	-30	N/A	N/A	10^5	9821	N/A	single crystal	3
BBDTY			-30	N/A	N/A	4429	9336	N/A	thin films	
BOPAnt	0.11	350	-60	N/A	N/A	4.34×10^5	3100	N/A	single crystal	4
C8-BTBT + PLA	0.02	365	-30	40 s	80 s	1×10^5	56	N/A	films (spin-coating)	5
DTTQ+ $CsPbBr_3$ QD	0.0005	365	60	0.114 s	0.118 s	1.8×10^4	7.1×10^5	3.6×10^{13}	films (solution-shearing)	6
1,6-DTEP	0.0002	370	-60	N/A	N/A	1.60×10^5	2.86×10^6	1.49×10^{18}	single crystal	7
2,7-DTEP			-60	N/A	N/A	4.35×10^3	1.04×10^5	5.28×10^{16}		
$PC_{61}BM$	0.007	315-400	80	0.94 ms	1.02 ms	9×10^3	3×10^3	1.3×10^{13}	films (spin-coating)	8
NDI-C_6+C_8-BTBT	0.3	365	-10	4 ms	6 ms	50	1.78	N/A	organic crystalline	9
2-An-BTBT	0.0177	380	-40	20 s	20 s	1576	7136	N/A	single crystal	10
TPA-An	0.0002	370	-60	N/A	N/A	1.03×10^3	7.19×10^5	1.40×10^{16}	single crystal	11
TBA-An			-60	N/A	N/A	3.45×10^4	1.50×10^5	1.60×10^{17}		
C8-BTBT+ polythioether	4.0	350	-30	21 s	16 s	1.0×10^5	2.5	6.3×10^{14}	films (spin-coating)	12

**NDI-PM**	0.001	365	100	229 ms	268 ms	2.0 × 10^5	1980	1.8 × 10^{14}	thin-films	13
**BTBTT6-syn (Bare SiO_2)**	3.1× 10^{-5}	370	-60	N/A	N/A	1.2 × 10^4	9 × 10^6	2 × 10^{17}	thin-films	This work
**BTBTT6-syn (SiO_2/PS)**	3.1× 10^{-5}	370	-60	N/A	N/A	1.64 × 10^5	4.5 × 10^6	3.9 × 10^{16}		
**BTBTT6-syn (SiO_2/PMMA)**	3.1× 10^{-5}	370	-60	N/A	N/A	3.47 × 10^5	1.2 × 10^6	7.1 × 10^{16}		
**BTBTT6-syn (SiO_2/PPO)**	3.1× 10^{-5}	370	-60	N/A	N/A	8 × 10^5	3.6 × 10^6	2.1 × 10^{17}		

Supplementary Note 1

In convolution processing, a matrix of weight values used to perform a weighted average operation is designed and called convolution kernel. Convolution kernel can be used in the convolutional neural network to extract local features after training[14]. In this case, the OPT array is used as a convolution kernel for extracting UV feature. It is worth noting that such convolution kernel is used to perform a weighted average operation on the RGB and UV values of a single pixel. Therefore, a device array with the same size as the input image can realize the whole convolution processing in one step. A weight function based on device characteristics is defined and would be used in subsequent computer simulation. Since the device exhibits wavelength-dependent tunable synaptic plasticity, the result of convolution processing based on the device characteristics is the sum of the responses to light of different wavelengths. The equation describing the convolution operation is as follows:

$$f_{output} = \frac{f_R(\mathrm{R})+f_G(\mathrm{G})+f_B(\mathrm{B})+f_{UV}(\mathrm{UV})}{4} \quad (1)$$

where f_{Output} is the output value obtained by weighting and averaging the RGB and UV values of a pixel. $f_R(R)$, $f_G(G)$, $f_B(B)$ and $f_{UV}(UV)$ are the weight functions of the R, G, B and UV values in the image, respectively. Corresponding to the device characteristics, the output is the sum of the current change in response to light spikes at 650 nm, 520 nm, 450 nm and 370 nm. Under the irradiation of different light wavelengths. As shown in Figure S19 and S20, the EPSC and weight update values

obtained under the illumination of RGB light are much lower than that of UV light, that is, the information given by the UV light is the dominate information. It is assumed that the output gray value of every pixels of RGB in the image are approximated by the ratio of the weight values between RGB and UV.

To demonstrate the advantages in image recognition with the pre-processing feature of focusing on UV information, the comparations of image recognition accuracy under different conditions are needed. In this case, three test datasets are used. The first dataset is the initial MNIST test dataset, including 10,000 test images (where each image is 20 × 20 pixels). RGB mode are used to store information with different colors in the images, and a separate value is introduced to storage the UV information. For the second dataset, the Gaussian noise is added into the RGB values and the UV information is not changed, so that the images with fuzzy visible light information and clear UV information are obtained. Then, the third dataset is gained with the second dataset pre-processed by the simulated convolution kernel array, in which the UV information is strengthened and the visible light information is weakened. So, the third dataset simulated the situation that vision system focusing on the UV information.

The image recognition process is performed using a three-layer ANN (400 input neurons, 100 hidden neurons and 10 output neurons). The activation functions of the hidden layer and output layer are Relu and Softmax, respectively. The synaptic weight (*w*) in the ANN are determined by a pair of normalized conductance values:

$$w = \frac{G^{+} - G^{-}}{G_{max}} \tag{2}$$

The recognition accuracy is calculated using the experimental LTP/LTD data (Figure S21), the recognition accuracy of each dataset is calculated by:

$$Accuracy\ (\%) = \frac{n}{\mathrm{N}} \times 100\% \tag{3}$$

where Accuracy and n denote the recognition accuracy and the number of correctly identified images, respectively. N is the total number of images in the dataset, equal to 10,000.

Supplementary Note 2

As far as we know, at present, the reported papers proposed several methods for dealing with the

"ghost shadow" problem, including hardware and software. Among which, the usage of software fusion algorithm is more common. In this case, the swinging lilies are used to simulate the process for the elimination of "ghost shadow" by the BTBTT6-syn-based devices. To solve the ghosting in the results caused by the moving objects in both images, we introduce the separation ratio as the proportion of separation between the images of swinging lilies before and after moving. The dynamic image pre-processing is achieved with introducing the interframe differential computations (Ref. 15). In the program, by calculating the differences between the UV perceived and stored image brightness at t and $t+\Delta t$ moment, the contours of the moving objects in the initial and final images can be clearly distinguished. When setting t and Δt to be the frame 41 and 16 frames (i.e., Δt =360 ms, Figure 5d), the separation ratio would reach to 100%.

References

1. Luo, L. et al. Charge-transfer pentacene/benzothiadiazole derivative cocrystal for UV-to-NIR large range responsive phototransistors. *Org. Electron.* **100**, 106363 (2022).
2. Jiang, L., Huang, H., Gui, F., Xu, Y. & Lin, Q. Ultrasensitive UV-NIR broadband phototransistors based on $AgBiS_2$–organic hybrid films. *J. Mater. Chem. C.* **9**, 7583–7590 (2021).
3. Zhao, G. et al. High-performance UV-sensitive organic phototransistors based on benzo[1,2-b:4,5-b′]dithiophene dimers linked with unsaturated bonds. *Adv. Electron. Mater.* **1**, 1500071 (2015).
4. Li, A. et al. Highly responsive phototransistors based on 2,6-bis(4-methoxyphenyl)anthracene single crystal. *J. Mater. Chem. C.* **5**, 5304–5309 (2017).
5. Huang, J. et al. Printable and flexible phototransistors based on blend of organic semiconductor and biopolymer. *Adv. Funct. Mater.* **27**, 1604163 (2017).
6. Hong, S.H. et al. Photoelectric effect of hybrid ultraviolet-sensitized phototransistors from an n-type organic semiconductor and an all-inorganic perovskite quantum dot photosensitizer. *Nanoscale* **13**, 20498–20507 (2021).
7. Tao, J. et al. Organic UV-sensitive phototransistors based on distriphenylamineethynylpyrene derivatives with ultra-high detectivity approaching 10^{18}. *Adv. Mater.* **32**, 1907791 (2020).

8. Huang, W., Lin, Y.H. & Anthopoulos, T.D. High speed ultraviolet phototransistors based on an ambipolar fullerene derivative. *ACS Appl. Mater. Interfaces* **10**, 10202–10210 (2018).

9. Guo, J. et al. Few-layer organic crystalline van der waals heterojunctions for ultrafast UV phototransistors. *Adv. Electron. Mater.* **6**, 2000062 (2020).

10. Yan, L. et al. Investigating the single crystal ofet and photo-responsive characteristics based on an anthracene linked benzo[b]benzo[4,5]thieno[2,3-d]thiophene semiconductor. *Org. Electron.* **72**, 1–5 (2019).

11. Tao, J. et al. Organic single crystals with high photoluminescence quantum yields close to 100% and high mobility for optoelectronic devices. *Adv. Mater.* **33**, 2105466 (2021).

12. Peng, H. et al. Interface engineering via photopolymerization-induced phase separation for flexible UV-responsive phototransistors. *ACS Appl. Mater. Interfaces* **10**, 7487–7496 (2018).

13. Song, I. et al. High-performance visible-blind UV phototransistors based on n-type naphthalene diimide nanomaterials. *ACS Appl. Mater. Interfaces* **10**, 11826–11836 (2018).

14. Li, G. et al. Photo-induced non-volatile VO_2 phase transition for neuromorphic ultraviolet sensors. *Nat. Commun.* **13**, 1729 (2022).

15. Zhang, Z.H. et al. All-in-one two-dimensional retinomorphic hardware device for motion detection and recognition. *Nat. Nanotechnol.* **17**, 27 (2022).
