# Supplementary material for: Tetrachromatic vision-inspired neuromorphic sensors with ultraweak ultraviolet detection
Source: Nat Commun. 2023 Apr 21;14:2281. doi: 10.1038/s41467-023-37973-0 (PMC10121588; doi:10.1038/s41467-023-37973-0)
Supplement: Supplementary file 3 — Description of Additional Supplementary Files [file 41467_2023_37973_MOESM3_ESM.docx]

File Name: Supplementary Movie 1

Description: The initial swinging lilies with petals and pistils.

File Name: Supplementary Movie 2

Description: After sensing with the device, the petal and pistil signals of swinging lilies were suppressed and enhanced respectively.

File Name: Supplementary Movie 3

Description: The motion detection result of swinging lilies with the interframe differential computations.
